# Altered Endothelin Receptor Expression in Idiopathic Male Infertility: A Potential Therapeutic Target

**DOI:** 10.5152/tud.2025.24104

**Published:** 2025-05-21

**Authors:** Richard Weiten, Melanie V. Brandenstein, Manuel Huerta, Tim Nestler, Axel Heidenreich, Enno Storz, Jan Herden

**Affiliations:** 1 Department of Urology, Uro-Oncology, Robot-Assisted and Specialized Urologic Surgery, University Hospital Cologne, Germany; 2 Department of Urology, Federal Armed Services Hospital Koblenz, Germany; 3 Department of Urology, Medical University Faculty of Medicine, Vienna, Austria; 4 Department of Urology, PAN Clinic Cologne, Germany

**Keywords:** Endothelin receptors, idiopathic male infertility, oligo-astheno-teratozoospermia, spermatozoa

## Abstract

**Objective:**

Idiopathic male infertility is frequently associated with impaired semen quality, particularly in men diagnosed with oligo-astheno-teratozoospermia (OAT) syndrome. Recent studies suggest a potential role of the endothelin (ET) system, particularly ET receptors A (ETAR) and B (ETBR), in male reproductive physiology. Moreover, antiestrogens such as tamoxifen and clomiphene, which are commonly used empirically in this context, may influence ET signaling pathways. However, the relationship between ET receptor expression and specific subtypes of male infertility remains poorly understood.

**Methods:**

Semen samples were collected from 56 men: 15 fertile controls with normozoospermia and 41 infertile men with abnormal semen parameters, including 22 with OAT syndrome and 19 with isolated teratozoospermia. Men with identifiable female-factor infertility were excluded. Seminal ETAR and ETBR expression were analyzed using immunofluorescence microscopy, Western blotting, enzyme-linked immunosorbent assay (ELISA), and flow cytometry.

**Results:**

The ETAR expression was significantly reduced in infertile men compared to fertile controls (*P* < .05). The ETBR was undetectable in all samples from patients with OAT syndrome. Notably, 2 distinct ETAR expression profiles were observed within the OAT group: one subgroup with ETAR levels comparable to controls and another with markedly diminished expression, indicating potential phenotypic heterogeneity within OAT syndrome.

**Conclusion:**

Altered ETAR expression and the absence of ETBR in men with idiopathic infertility, particularly those with OAT syndrome, highlight a potential role for ET signaling in male reproductive dysfunction. Stratification based on ETAR expression may support individualized therapeutic strategies, including ET-targeted or antiestrogen-based therapies, to improve fertility outcomes in this population.

**Main Points**

- Endothelin (ET) receptor expression is altered in the semen of infertile men diagnosed with oligo-astheno-teratozoospermia (OAT) syndrome.
- Two distinct ETAR expression patterns were identified within the OAT subgroup, indicating underlying biological heterogeneity.
- Personalized therapeutic strategies targeting specific ET receptor profiles may offer new avenues to improve sperm quality in idiopathic male infertility.

## Introduction

Infertility is a growing globalhealth concern, affecting a substantial number of couples, with its prevalence increasing steadily over recent decades.^1^^,^^2^ Male factors are implicated in approximately 20%-70% of infertility cases, with the global prevalence of male infertility estimated to range between 2.5% and 12%, affecting more than 30 million men worldwide.^2^ One of the most common diagnostic categories among infertile men is oligo-astheno-teratozoospermia (OAT) syndrome, which is defined by the concurrent presence of oligozoospermia (low sperm concentration), asthenozoospermia (reduced motility), and teratozoospermia (abnormal morphology).^1^^-^ ^3^ Although various genetic, endocrine, and environmental factors have been proposed, the underlying etiology of OAT syndrome often remains idiopathic.^2^^,^^3^

Empirical medical therapies, particularly antiestrogens such as tamoxifen and clomiphene citrate, are commonly used in the treatment of idiopathic male infertility.^4^^,^^5^ These agents exert their effects by antagonizing estrogen receptors in the hypothalamus and pituitary gland, leading to increased secretion of gonadotropin-releasing hormone, which stimulates luteinizing hormone (LH) and follicle-stimulating hormone (FSH) production. This cascade enhances Leydig cell-mediated testosterone synthesis and supports Sertoli cell function, ultimately promoting spermatogenesis.^6^

Although early studies reported only modest improvements in pregnancy rates with antiestrogen therapy,^5^^,^^7^^,^^8^ more recent meta-analyses, including 11 randomized trials, have demonstrated significant improvements in both sperm parameters and pregnancy rates among men with idiopathic infertility, particularly those with OAT syndrome.^4^^,^^5^^,^^7^ Nevertheless, the clinical utility of antiestrogens remains debated due to heterogeneity in patient selection and variable treatment responses.

Recent research has highlighted the potential role of the endothelin (ET) system in male reproductive physiology. Endothelin-1 (ET-1), a potent vasoconstrictive peptide, has been detected in human seminal plasma, with lower concentrations observed in men with OAT syndrome compared to normozoospermic controls.^9^^-^ ^11^ Moreover, antiestrogens such as tamoxifen have been shown to upregulate ET-1 and its receptors in other biological systems, including breast cancer tissue.^11^ Conversely, the use of ET receptor antagonists (e.g., bosentan) in animal models has been associated with testicular atrophy, reduced sperm production, and impaired fertility, suggesting that intact ET signaling is necessary for normal spermatogenesis.^9^^-^ ^11^

These findings suggest a mechanistic link between antiestrogen therapy and ET signaling, with potential implications for male fertility. In particular, ET receptors A (ETAR) and B (ETBR) may serve as downstream effectors or modulators of treatment response. Based on this rationale, it was hypothesized that altered expression of ETAR and ETBR may underlie idiopathic infertility, particularly in men with OAT syndrome, and that characterization of receptor expression patterns could inform treatment stratification. The present study aimed to systematically evaluate the expression of ETAR and ETBR in semen samples from men with idiopathic infertility, including those with OAT syndrome and isolated teratozoospermia, compared to normozoospermic controls.

## Material and Methods

### Patients’ Samples

Semen samples were collected from 56 men, including 15 fertile controls (normozoospermic) and 41 infertile patients with abnormal semen parameters, comprising 22 with OAT syndrome and 19 with isolated teratozoospermia. Semen analysis and categorization were performed according to the World Health Organization (WHO) laboratory manual for human semen analysis.^12^^,^^13^

Inclusion criteria were age ≥18 years and the availability of complete clinical and semen analysis data. Fertile controls were defined as men with documented paternity within the past 2 years and normozoospermic profiles based on WHO reference values. Infertile patients had a history of at least 12 months of unprotected intercourse without conception and met criteria for OAT syndrome or isolated teratozoospermia.

Exclusion criteria included a history of urogenital disorders (e.g., varicocele, orchitis, testicular torsion, malignancy), prior exposure to chemotherapy, radiotherapy, or hormonal therapy, known genetic causes of infertility (e.g., Klinefelter syndrome), systemic illnesses, current infections, and incomplete clinical or laboratory data.

Participants abstained from ejaculation for 2-7 days before sample collection. The study was approved by the Ethics Committee of the Medical Faculty of Medical Faculty of the University of Cologne (Approval No. 22-1178 Date: 18.10.2022) and registered in the German Clinical Trials Registry (DRKS00029986). Written informed consent was obtained from all participants.

### Immunofluorescence

Semen samples were centrifuged at 2500 × g for 5 minutes and washed twice with phosphate-buffered saline (PBS; PAN-Biotech GmbH, Germany). A 10 µL aliquot was applied to glass slides (Thermo Fisher Scientific, Germany) and incubated with primary antibodies against ETAR (#sc-33536) and ETBR (#sc-518149; both from Santa Cruz Biotechnology, USA) at 1 : 1000 dilution for 1 hour. Fluorescently labeled secondary antibodies (FITC- or Alexa 594-conjugated anti-mouse IgG; Santa Cruz Biotechnology) were applied for 30-60 minutes. Nuclei were counterstained using DAPI-containing mounting medium (Abcam, UK). Images were captured using an Olympus DP7 fluorescence microscope and analyzed using DISCUS software.

### Western Blotting

Sperm lysates (1 × 10^6^ cells) were prepared using RIPA buffer (Biomol, Germany) supplemented with protease inhibitors. Protein concentrations were determined with the Pierce BCA Protein Assay Kit (Thermo Fisher Scientific, USA). Samples were denatured at 95°C in 4 × SDS loading buffer, separated by SDS-PAGE (4% gel), and transferred to 0.45 µm nitrocellulose membranes (Merck, Germany). Membranes were blocked with 5% non-fat milk in TBST and incubated overnight at 4°C with primary antibodies against ETAR, ETBR (both 1 : 500), and GAPDH (1 : 1000; all from Santa Cruz Biotechnology). HRP-conjugated anti-mouse secondary antibodies (Thermo Fisher Scientific, 1 : 5000) were used, and bands were visualized using chemiluminescence (ChemoStar ECL Imager, INTAS, Germany).

### Enzyme-Linked Immunosorbent Assay

Enzyme-linked immunosorbent assay was performed as previously described.^14^ Samples were diluted to 6 × 10^5^ cells per 50 µL. The ETAR and ETBR protein levels were quantified using primary antibodies (Santa Cruz Biotechnology, 1 : 1000) and detected via fluorescent signal using the FLUOstar Omega® microplate reader (BMG Labtech, Germany).

### Flow Cytometry

Flow cytometric analysis was conducted using standard immunostaining protocols. Semen aliquots (50 µL) were incubated with anti-human Vim3 (Davids Biotechnologie GmbH, Germany) and Mxi-2 (nanoTools, Germany) primary antibodies (1 : 500). Detection was performed using Alexa 488-conjugated anti-mouse secondary antibodies (Santa Cruz Biotechnology). Data were acquired using the Attune CytPix flow cytometer (Thermo Fisher Scientific, Germany) and analyzed with FlowJo® software (version 10.8, BD Biosciences).

### Statistics

Statistical analysis was performed using GraphPad Prism® software (version 9.4.0, GraphPad Software, San Diego, CA, USA). Group comparisons were assessed using the Kruskal–Wallis test. All tests were two-tailed, and a *P*-value < .05 was considered statistically significant.

## Results

### Study Cohort

Semen samples from 56 men were included: 15 fertile controls with normozoospermia and 41 infertile patients subdivided into 22 with OAT syndrome and 19 with isolated teratozoospermia. The classification allowed for phenotype-specific analysis of ET receptor expression. Demographic and semen parameter data are summarized in Table 1.

### Immunofluorescence Localization of Endothelin Receptors

**Figure 1.**
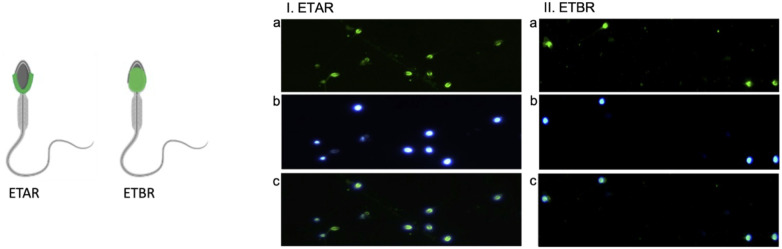
Immunofluorescence staining of endothelin receptors in normozoospermic semen. I: ETAR expression: I.A, ETAR expression (green) is localized to the plasma membrane surrounding the post-acrosomal region of the sperm head. I.B, Sperm DNA (blue, stained with DAPI) is confined to the head. I.C, the merged image confirms colocalization of ETAR and DNA signals in the head region. II: ETBR expression: II.A, ETBR expression (green) is observed throughout the post-acrosomal area of the sperm head. II.B, DAPI staining (blue) highlights the sperm nucleus. II.C, the merged image demonstrates overlapping localization of ETBR and nuclear staining. Abbreviations: DAPI, 4’,6-diamidino-2-phenylindole, ETAR, endothelin receptor type A; ETBR, endothelin receptor type B.

In normozoospermic controls, immunofluorescence revealed localization of ETAR and ETBR to the sperm head. The ETAR was primarily detected along the plasma membrane adjacent to the post-acrosomal region, whereas ETBR displayed a more diffuse distribution across the post-acrosomal area (Figure 1).

### Quantification of Endothelin Receptor Expression

**Figure 2.**
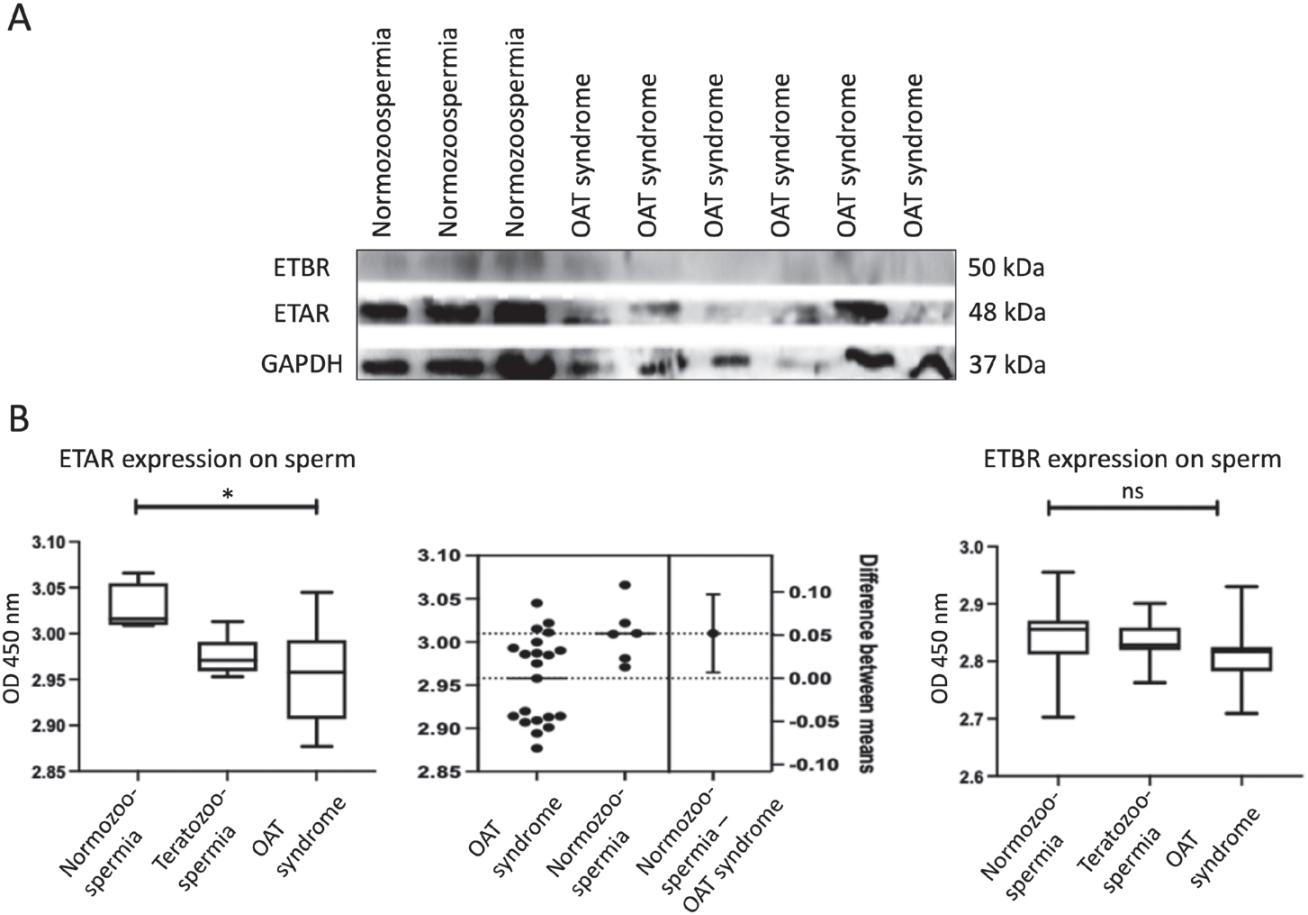
Protein expression levels of endothelin receptors in semen samples. ETAR and ETBR protein expression levels in semen samples were assessed using Western blotting (A) and ELISA (B) in both fertile (normozoospermia) and infertile males (with OAT syndrome and teratozoospermia). A, Strong ETAR expression was found in normozoospermic controls and in a subset of patients with OAT syndrome, whereas ETBR was undetectable in all samples. Detection of GAPDH served as a loading control. B, Increasing ETAR expression levels in semen of fertile (normozoospermia) men compared to infertile participants (Kruskal-Wallis test, p < 0.05). ETBR expression showed no significant differences between groups. Two distinct ETAR expression patterns were identified within the OAT group. Abbreviations: ETAR, endothelin receptor type A; ETBR, endothelin receptor type B; OAT syndrome, oligo-astheno-teratozoospermia.

Western blotting and ELISA confirmed reduced ETAR expression in infertile patients compared to controls (*P* < .05; Kruskal–Wallis test). While ETAR levels varied among OAT patients, 2 distinct expression subgroups emerged: one with ETAR levels similar to controls, and another with markedly reduced expression (Figure 2A-B). The ETBR was undetectable in OAT samples and did not show significant intergroup variation by ELISA. (Figure 2B).

### Flow Cytometry Validation

**Figure 3.**
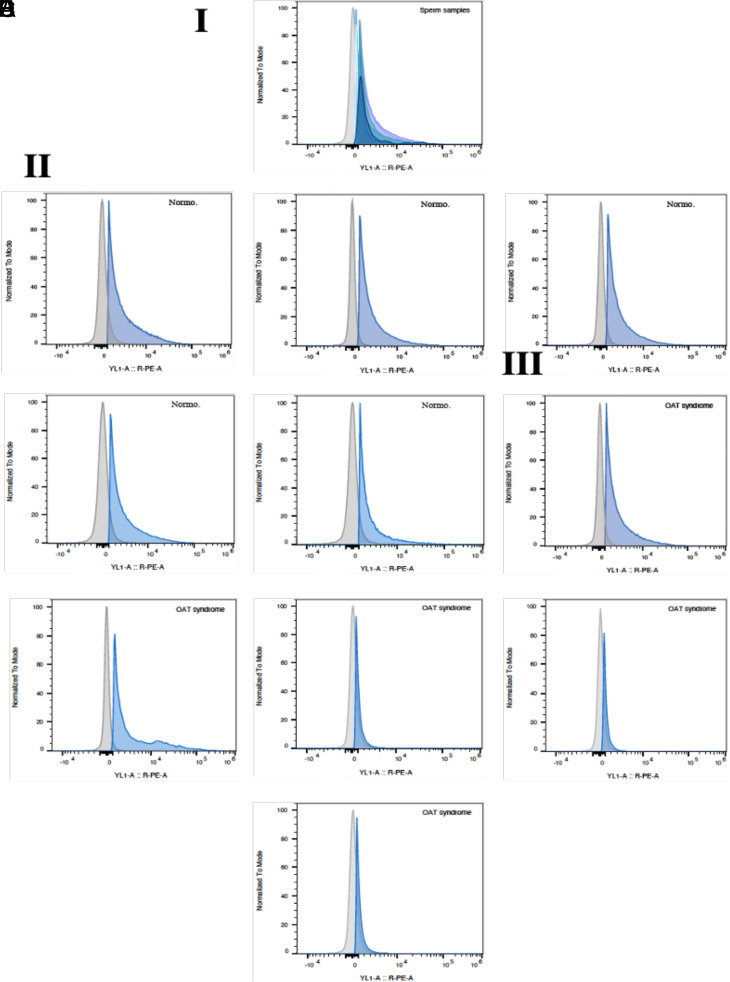
Flow cytometry analysis of ETAR expression levels in semen samples. I, Normalized histogram showing ETAR expression (blue peak) as detected by flow cytometry in semen samples from 5 normozoospermic patients and 5 patients with OAT syndrome. An unstained control (grey peak) was included as a reference. IIa-e: Representative flow cytometry profiles from normozoospermic individuals demonstrating consistently high ETAR expression across all 5 samples. IIIa-e: Flow cytometry profiles from OAT patients. Samples IIIa and IIIb exhibited ETAR expression levels comparable to those of normozoospermic controls, whereas samples IIIc–e showed markedly reduced ETAR expression. Abbreviations: ETAR, endothelin receptor type A; OAT, oligo-astheno-teratozoospermia.

Flow cytometry of samples from 5 controls and 5 OAT patients corroborated prior findings. The ETAR expression was higher in fertile men and showed a bimodal distribution among OAT patients, confirming the presence of 2 phenotypically distinct ETAR expression subgroups within the OAT cohort (Figure 3).

## Discussion

This study is the first to systematically investigate the expression patterns of ETAR and ETBR in distinct subgroups of men with idiopathic infertility, specifically those diagnosed with OAT syndrome and isolated teratozoospermia. By stratifying these subgroups, it was aimed to determine whether ET receptor dysregulation is associated with global semen impairment, as seen in OAT, or whether it may also occur in the context of isolated morphological abnormalities.

The results demonstrate that ETAR expression is significantly reduced in both OAT patients and those with isolated teratozoospermia, suggesting that dysregulation of ETAR may occur independently of oligozoospermia or asthenozoospermia. Notably, while patients with isolated teratozoospermia exhibited relatively better sperm morphology than those in the OAT group (1.66% vs. 0.59%, respectively), their ETAR expression remained significantly lower than that of normozoospermic controls. This indicates that impaired morphology alone may be associated with altered ETAR expression and supports the hypothesis that ETAR dysregulation could contribute to infertility even in the absence of more pronounced abnormalities in sperm count or motility.

Previous studies have highlighted the role of ETAR in male reproductive physiology, particularly in regulating sperm motility and the acrosome reaction.^9^^-^ ^11^ The data build upon this foundation by revealing inter-individual variability in ETAR expression within the OAT cohort, with 2 distinct ETAR expression patterns. This heterogeneity may have clinical implications, as patients with preserved ETAR expression could potentially benefit more from empiric therapies such as clomiphene citrate or tamoxifen, agents known to influence the ET signaling pathway.^4^^-^ ^7^ Thus, ETAR profiling may serve as a basis for individualized therapeutic approaches in male infertility.

In contrast, ETBR expression was consistently undetectable in both OAT and isolated teratozoospermia groups. This observation aligns with preclinical models in which ETBR deficiency is associated with impaired spermatogenesis and compromised testicular function.^10^ The findings suggest that ETBR may play a limited or absent role in sperm function in the context of idiopathic male infertility, although further investigation is warranted.

A key strength of this study lies in its comprehensive, multimodal methodological approach. By integrating immunofluorescence, ELISA, Western blotting, and flow cytometry, a robust and cross-validated assessment of receptor expression was ensured. Additionally, the phenotypic stratification of infertile patients allowed for a more granular analysis of receptor patterns, uncovering differences that might otherwise be masked in more heterogeneous populations. Compared to previous studies, which often focused on animal models or normozoospermic individuals, the work provides novel insights into receptor expression specifically in human idiopathic infertility subtypes.

Taken together, the findings underscore the potential importance of the ET system, particularly ETAR, in the pathophysiology of idiopathic male infertility. Moreover, the identification of ETAR expression subtypes within the OAT population offers a possible avenue for biomarker-driven stratification and therapeutic targeting. Future studies are warranted to validate these findings and to investigate the mechanistic role of ETAR signaling in male reproductive function.

### Limitations

This study has several limitations. First, the sample size for flow cytometry was relatively small (n = 5 per group), which may limit the generalizability and statistical power of subgroup comparisons. Second, although strict inclusion criteria were applied to exclude known confounding factors, unmeasured variables such as environmental exposures, oxidative stress, or epigenetic alterations may have influenced ET receptor expression. Third, the investigation was limited to protein-level analysis; functional assays to assess receptor activity, downstream signaling pathways, or ligand interactions were not performed and would be necessary to establish causality. Fourth, receptor expression was evaluated only in ejaculated spermatozoa. Assessing ETAR and ETBR expression in testicular tissue, Sertoli cells, or seminal plasma could offer a more comprehensive understanding of the ET system’s role in spermatogenesis. Lastly, the cross-sectional design precludes evaluation of longitudinal changes in receptor expression or their potential predictive value in treatment response.

In summary, this study identifies distinct alterations in ET receptor expression in men with idiopathic infertility. Specifically, ETAR expression was reduced in both OAT syndrome and isolated teratozoospermia, suggesting that its dysregulation may contribute to male infertility independently of broader semen quality parameters. The detection of 2 ETAR expression profiles within the OAT subgroup highlights the biological heterogeneity of this condition and supports the potential value of ETAR as a biomarker for individualized treatment planning. The consistent absence of ETBR in infertile men suggests a limited role for this receptor subtype in mature sperm. Future research should include longitudinal studies, functional assays, and larger, well-characterized cohorts to evaluate the prognostic and therapeutic implications of ET receptor expression in male infertility.

## Figures and Tables

**Table 1.** Patient Age and Semen Analysis of Fertile and Infertile Males

	Fertile Males (n = 15) Normozoospermia (n = 15)	Infertile Males (n = 41)		*P*
		OAT Syndrome (n = 22)	Teratozoospermia (n = 19)	
Age (years) (Mean ± SD)	35.40 ± 6.34	35.23 ± 4.76	36.89 ± 5.14	.574
Semen volume (mL) (Mean ± SD)	2.45 ± 1.04	2.80 ± 1.90	4.05 ± 1.55	.01
Sperm concentration (10^6^/mL) (Mean ± SD)	92.60 ± 55.78	5.24 ± 4.10	56.26 ± 33.04	<.001
Total sperm count (10^6^ cells) (Mean ± SD)	233.17 ± 180.34	12.96 ± 13.76	206.16 ± 108.82	<.001
Progressive sperm motility (%) (Mean ± SD)	55.97 ± 12.54	20.18 ± 5.93	50.13 ± 10.12	<.001
Normal sperm morphology (%) (Mean ± SD)	7.07 ± 2.95	0.59 ± 0.81	1.66 ± 0.96	<.001
Serum T (ng/mL) (Mean ± SD)	3.96 ± 1.45	4.37 ± 1.44	4.53 ± 1.62	.539
Serum LH (mIU/mL) (Mean ± SD)	3.23 ± 0.94	5.23 ± 1.83	3.38 ± 1.49	.02
Serum FSH (mIU/mL) (Mean ± SD)	3.86 ± 1.37	9.51 ± 6.22	5.05 ± 2.57	.001
Serum PRL (ng/mL) (Mean ± SD)	7.89 ± 3.79	10.77 ± 6.49	9.27 ± 2.85	.207

FSH, follicle-stimulating hormone; LH, luteinizing hormone; OAT syndrome, oligo astheno-teratozoospermia; PRL, prolactin; SD, standard deviation; T, testosterone.

## Data Availability

The data that support the findings of this study are available from the corresponding author upon reasonable request.
